# The symptomatic management of multiple sclerosis

**DOI:** 10.4103/0972-2327.58278

**Published:** 2009

**Authors:** Randall T. Schapiro

**Affiliations:** The Schapiro Multiple Sclerosis Advisory Group and Clinical Professor of Neurology, University of Minnesota, USA

**Keywords:** Management, multiple sclerosis, symptom

## Abstract

The management of multiple sclerosis (MS) revolves around disease management, symptom management, and person management. Of these, symptom management takes up the bulk of the time of the practicing physician. Some symptoms are easily managed whereas others are more difficult. Decisions have often to be made on whether to treat or to wait and watch. This article discusses the varied symptoms of MS and the approaches to management, which involves rehabilitation, pharmacological treatments, and surgical procedures. The skilled physician managing MS should be familiar with the multiple approaches to improving the quality of life of those with MS. After the diagnosis has been established and the decisions regarding treatment approaches have been made, the talk in a typical office appointment for MS usually turns to symptom management. Thus, the majority of management decisions made by the clinician revolve around that important topic. It is symptom management that will determine quality of life for those with MS, It is the basis for improving function, and, up until twenty years ago, it was the only basis for treating MS. Now, however, we can approach treatment by disease management, symptom management, and person management. The MS specialist must be well versed in all three areas.

The symptoms of MS have traditionally been divided into three categories: 1) primary symptoms are those that occur because of actual demyelination within the central nervous system; 2) secondary symptoms are those that emanate from the primary symptoms, e.g., spasticity leads to contractures, bladder dysfunction leads to infections, etc.; 3) tertiary symptoms are those that occur because of psychological reactions to the stresses (vocational, personal, or marital problems) associated with chronic disease.

For the purposes of this paper, I have chosen a different approach. Some symptoms associated with MS are usually easily treatable, some are harder to treat, and some are very difficult to treat. I will use this classification when I discuss the management of the different symptoms.

The ‘tools’ used to treat symptoms, in general, fall into three broad categories: 1) rehabilitation, 2) pharmacological, and 3) procedural. Some of these areas are covered in other papers within this set of articles. It is important to understand that none of these techniques needs to stand alone unless it is by itself sufficient to remedy the particular symptom/problem. Usually, however, a combination of tools is necessary to ease the various symptoms.

Urinary problems secondary to a dysfunctional bladder are very common in MS.[1] The symptoms of urgency, frequency, hesitancy, and incontinence indicate unregulated nerve function somewhere along the spinal axis, from the brain to the sacral cord. Localization of the lesion is impractical and almost impossible.[2] The symptoms of a small, hypertonic, ‘failure-to-store’ bladder may be the same as those of a large, hypotonic, ‘failure-to-empty’ bladder.[3] The treatment, however, is very different. To treat appropriately, the clinician must first determine what kind of a bladder is most likely to be present in that particular individual. Eliminating any contributing infection is a necessity. Upper urinary tract (kidney and ureter) infection is surprisingly not common in MS, but bladder infections are very common.[4] Controlling bladder function in the face of infection is almost impossible; thus, if infection is found, antibiotics and other bladder cleansing techniques (acidification) should first be instituted. Following that, measurement of residual urine will give a quick idea as to whether the bladder is small and contracting frequently or large and overflowing (both conditions give rise to the very same symptomatology). Measurement of residual urine can be done via catheterization or via ultrasonography.

Physical therapists who are trained in pelvic floor rehabilitation can instruct those with a small bladder to help decrease frequency and urgency.[5] It takes a skilled therapist and a motivated patient to benefit fully from these techniques, but it is certainly possible.

When the bladder is found to be small (i.e., there is low residual urine volume), anticholinergic medication will usually decrease the urgency and frequency.[6] There are many anticholinergic medications available, depending on the regional formularies (e.g., oxybutynin, tolteridine tartrate, solifenacin, darifenacin, and hyoscyamine). The dose will need to be increased till maximal effectiveness is reached; the clinician must remember that sweating is retarded with anticholinergic treatment and that this may cause the internal body temperature to rise, which may actually worsen the initial symptoms.

If the residual urine is high (over 150 cc), the use of agents that increase flow would seem logical. Alpha-adrenergic blockers and agents used for treatment of prostatic obstruction (e.g., tamsulosin, terazosin, and prozosin) are often utilized but with little reported success.[7]

Catheterization techniques may work well, if mastered. Intermittent self-catheterization is practical if the person has reasonable sensation and coordination of the hands. It is clean (not sterile) and, by reducing the amount of retained urine, the infection rate actually decreases.[8] Bladder-slowing anticholinergic medication may then be added to reduce the frequency of catheterizations if necessary. If there is poor coordination in the upper extremities, a Foley catheter with continuous drainage may be necessary. While the infection rate may be somewhat higher with this catheter, the quality of life can be improved dramatically and that should not be ignored.

There are situations in the MS bladder when the sphincter goes into spasm even as the bladder contracts. This dyssynergia results in urgency, hesitancy, and leakage.[9] The degree of dyssynergia can be measured by urodynamic testing and if improvement in bladder function cannot be achieved by the above methods, this testing may provide the answer. Dyssynergia is of special concern in the male as it may lead to high bladder pressure forcing urine up the ureters to the kidneys. While this may be a problem in the female, anatomy decreases its likelihood.

Urologists often take little interest in the MS bladder as there are few procedures that they can do to help. However, if there is an overactive bladder that does not respond to oral agents, botulinum toxin injected into the bladder wall will decrease its hyperactive state.[10] Electrical stimulation of the sphincter may help in some problematic situations but this technique is still experimental in the MS bladder.[11]

For most MS patients the bowel is less of a problem than the bladder. However, if problematic, it can cause much embarrassment. Constipation is more common than diarrhea. Often this is due to a sluggish bowel secondary to physical inactivity and decreased fluid intake. The decreased fluid intake may be the result of the patient's attempt to decrease bladder symptoms by fluid restriction. Fiber and fluid are the basis of a good bowel program.[12] Understanding that the gastrocolic reflex stimulates defecation about 30 min following a meal is helpful.[13] Utilizing stool softeners and fiber laxatives, along with the occasional suppository, is helpful. Enemas are a last resort but may be necessary from time to time to avoid obstruction and to achieve better timing and control of bowel movement and to avoid incontinence of stool.

Spasticity is a common and treatable symptom in MS.[14] It is a velocity-dependent stiffness, with increase in muscle tone when the limb is moved quickly. Not all spasticity needs treatment. However, if it gets in the way of function or is painful, it should be managed.[15] To decrease the muscle tone secondary to spasticity, noxious stimuli should be removed, as pain anywhere in the body will increase spasticity.[16] Following this, a rigorous stretching program with range-of-motion exercises, accompanied by some aerobic exercise, should be instituted.

Medication is usually effective for relieving spasticity.[17] These neurochemical modulators are dose-titrated to get the most benefit. Baclofen, tizanidine, dantrolene, clonazepam, and a number of other such drugs may be given in combination to decrease tone.[18] If pharmacotherapy fails, a more aggressive approach may be needed. For example, botulinum toxin may be injected into the spastic muscle to decrease muscle tone;[19] this can be an excellent approach when there is spasticity of a single muscle. If limb spasticity, involving several muscles in an extremity is present, an intrathecal baclofen pump delivering baclofen directly into the central nervous system is usually effective.[20] The drug is delivered via a programmable pump that allows dosage adjustment. If this fails, neurosurgical intervention via a rhizotomy can be done, but this is rare in today's management schemata.

There are a number of conditions with paroxysmal symptoms that respond to anticonvulsant treatment.[21] Trigeminal neuralgia usually responds to carbamazepine and a number of other anticonvulsants. Paroxysmal spasms of the extremities may occur several times a minute and often appear mysterious to the inexperienced clinician but, again, are easily managed once they are recognized. Electric sensations down the spine (LHermitte's sign) are similarly medicated if bothersome.

A number of symptoms are harder to treat. Fatigue is the most common symptom seen in MS and is often difficult to treat.[22] There are five distinct types of fatigue in MS. 1) Normal fatigue is easy to understand (although the patient may not recognize it as normal). Occupational therapists may help with the activities of daily living and teach the patient to organize the day better. This can help in normal fatigue as well as in the other varieties. 2) ‘Short-circuiting' fatigue is also easy to understand. The demyelinated nerve fires until it blocks and the result is neuromuscular fatigue and weakness. It is treated with rest and cooling. 3) The fatigue of deconditioning is understandable, if recognized, and may be helped by a graded exercise program. 4) The fatigue of depression may be difficult to measure but, again, is treatable if recognized. 5) The most common fatigue is the one that is least understood; it has been labeled 'lassitude’ or ‘MS-related fatigue'.[23] This overwhelming tiredness is difficult to understand or measure but may be severely disabling. It is thought to have a neurochemical etiology, as neurochemicals such as modafinil are often helpful in relieving the symptoms. Other neurochemicals such as amantidine and a number of serotonin-specific release inhibitors may be of value.

Depression is common in MS.[24] It is more common than in most other neurological diseases, an indication of the neurochemical imbalance that results from CNS damage. Suicide rates are higher in this population. Appropriate treatment with antidepressants and counseling is usually necessary. Often patients do not want to recognize their depression and clinicians will try to ignore it, leading to undesired results.

Ambulation is affected in over two-thirds of those with MS.[25] Using appropriate tools, e.g., canes, crutches, braces, or walkers, will keep people mobile longer and with less energy drain. Thus, the appropriate device should be prescribed early and its proper use should be taught. The answer to disability is mobility, and keeping people mobile should be the first and foremost thought. Mobility devices such as scooters and wheelchairs are helpful for all with limited mobility, even for those with some ambulation,.

Dizziness and vertigo come in many forms. They may present acutely and be severe, requiring steroid therapy, or may linger in a chronic form. Benzodiazepines and antihistamines may be helpful but often do not provide complete relief.[26] Vestibular rehabilitation, taught by a skilled physical therapist, is the best strategy for intractable vertigo.[27]

Sexual dysfunction is difficult to manage, mainly because it is not openly discussed. There are many strategies to combat erectile dysfunction.[28] Oral medication is very popular. Prostaglandin injection into the shaft of the penis prior to intercourse often succeeds. A penile prosthesis is an alternative when all else fails. There appears to be no answer for ejaculatory problems. For the female, the strategies are less defined. Sensory disturbance may be the issue. Vibrators or other stimulating devices (e.g., eros device or a frozen bag of peas) can be helpful. A decreased libido usually accompanies the disease process and may be worsened by medications (e.g., antidepressants) given for other symptoms.[29] There appears to be no clear solution for this all too common symptom.

Weakness secondary to CNS dysfunction is very difficult to treat. Often progressive resistive exercise advised as treatment leads to increased fatigue and increased weakness.[30] However, if the muscle is not used it will lose strength. Thus the exercise program must be tailored to the individual. In any case, improving strength remains a difficult problem. The aminopyridine potassium-channel blockers appear to have a potential to help with this process.[31] By allowing for increased nerve conduction, strength may be improved and, with that, function too. It is too early to say what the long-term effect of the availability of better standardized preparations will be. The generic forms of aminopyridines appear to be erratically absorbed and, besides, lower the seizure threshold to unacceptable levels.

Pain occurs in over 50% of those with MS. Pain management has been significantly improved with the advent of the newer anticonvulsant medications such as gabapentin, pregabulin, lamotrigine, and many others, which are all effective for neuropathic pain.[32] Routine analgesics do not help much and may actually lead to increased problems due to chronic use. Orthopedic causes for the pain must be ruled out as the treatment would potentially be quite different. Even with all the improvements in pain management, it remains a difficult symptom.

Cognitive problems occur in over 50% of those with MS.[33] The etiology is easy to understand as there is demyelination and axonal loss involving the main transporting fibers of the brain. Add to that the newly emphasized gray matter destruction and the set up for memory, planning, and judgment issues are frequent. Short of replacing the damaged tissue there is little that actually works to fix the problem. Replacement technology is not available as yet, but peripheral memory devices, e.g., organizers and notebooks, can be of major help. Occupational therapists can develop programs of rehabilitation that will keep people active and minimize the loss as much as possible. Medications utilized in the treatment of Alzheimer dementia may be of slight help and, depending on the risk/benefit/cost ratios, are worth a try. Looking for mitigating factors such as depression and overmedication is important.

The introduction of immune-modulating treatments opened up a whole new area of symptom management: side effect management. Both glatiramer acetate and β-interferon produce skin reactions.[34] The skin reactions are somewhat variable and may change as the treatments continue. With glatiramer there is often an initial redness, which is irritative but not particularly severe. Often, this can be remedied by giving the injection deeper into the subcutaneous tissues. With time, and if the drug is injected sufficiently deep into the subcutaneous tissues, the redness tends to disappear but almost inevitably over the years the subcutaneous tissue atrophies and a lipodystrophy with lumps, bumps, and cavitations appear.[35] The skin itself toughens and patients have a hard time finding a place to inject the drug(s).

About 10% of those utilizing glatiramer will have a post-injection reaction, with shortness of breath, chest pain, sweating, and a feeling of doom and gloom. This severely symptomatic reaction lasts around 20 min but can linger for hours before abating spontaneously. It is important for patients to be well educated about this reaction so that they can avoid inappropriate emergency care and the costs associated with that. Reassurance is the best treatment, although at the time this may appear very conservative.

The interferons (both β1a and β1b) cause skin reactions that can be dramatic, but by initiating treatment with a less than therapeutic dose and escalating slowly the reactions can be minimized;[36] in some patients it may take over a month's time to get to the full dose with good tolerance. Flu-like symptoms are common at the initiation of treatment and may also be well managed with slow dose escalation.[36] While lipodystrophy occurs with interferon it is far less than with glatiramer, which may simply be because fewer injections are needed. Intramuscular interferon-β1a does not cause skin reactions but the flu-like symptoms may be exaggerated. Injection pain may be a factor with all injectables and may respond to an anesthetic cream or simply cooling/heating the area prior to injection. Blood counts and liver function tests need monitoring and may necessitate dose adjustment.

Natalizumab administration may lead to allergic reactions that will necessitate termination of treatment. Anaphylaxis occasionally occurs and the reduction of the immune system's normal defenses can lead to opportunistic infections.[37] Mitoxantrone has been associated with heart failure and leukemia.[38] Monitoring for these diseases is necessary with the use of ultrasonography, blood tests, and observation. Thus, managing symptoms is a challenge with each and every available treatment.

In general, when managing MS the philosophy should be to enable each patient to do as well as he/she can with whatever disability/symptom they have. That means treating the disease, the symptoms of the disease, and the person who has the disease. That is a philosophy of action. However, the important medical adage, *primum non nocere* (above all, do no harm) needs heeding. At times the aggressive clinician loses track of the combination of medications used to treat the myriad of symptoms. Patients want treatment for all that ails them and the clinician often prescribes accordingly. Metabolic schemes may decrease the potency of various treatments. Some treatments may decrease libido or cause depression; other treatments may have the unwanted effect of decreasing sweating and may thereby produce heat intolerance. All of these issues are often forgotten in the heat of battle.

Another issue the clinician faces in the symptomatic management of MS is that all symptoms are not necessarily the result of MS. Often numbness of the hands turns out to be due to carpal tunnel syndrome and not central demyelination. The astute clinician must be on the watch for the odd-appearing brain tumor or aneurysm that occurs in the patient who just happens to have MS. Thyroid and B_12_ deficiencies may occur more often in the patient with MS. Thus, it is necessary to look out for other medical illnesses when managing MS.

Increasing the quality of life through symptom management is worthwhile and patients will be forever grateful to the physician for approaching their issues with interest.
